# Efficacy and Tolerability of Topical Trifarotene in Managing Acne Vulgaris Among Malaysians: A Single Arm, Open Label, Observational Study

**DOI:** 10.1111/jocd.71058

**Published:** 2026-07-09

**Authors:** Jun Hui Goh, Tatt Quan Tan, Wen Chung Ho, Chi Fai Lee, Meroshini Sundaran, JiaXu An, Kang Nien How

**Affiliations:** ^1^ Faculty of Medicine and Health Sciences Universiti Putra Malaysia Serdang Selangor Malaysia; ^2^ Dermatology Unit, Department of Medicine, Faculty of Medicine and Health Sciences Universiti Putra Malaysia Serdang Selangor Malaysia; ^3^ Dermatology Unit, Department of Medicine, Hospital Sultan Abdul Aziz Shah Universiti Putra Malaysia Serdang Selangor Malaysia

**Keywords:** acne vulgaris, efficacy, Malaysia, retinoids, tolerability, trifarotene

## Abstract

**Background:**

Trifarotene is a new fourth‐generation topical retinoid that selectively binds to RAR‐γ receptors. However, the efficacy and safety data of trifarotene on Asians are lacking. Thus, we evaluated its real‐world efficacy and tolerability among Malaysians.

**Methods:**

This prospective, 12‐week observational study at Hospital Sultan Abdul Aziz Shah, Malaysia, involved participants with mild‐to‐moderate facial acne. Efficacy was assessed using absolute lesion counts, Investigator's Global Assessment (IGA), Post‐Acne Hyperpigmentation Index (PAHPI), and Cardiff Acne Disability Index (CADI). Tolerability was evaluated by using a modified safety assessment scale and participant‐reported side effects. Participants' satisfaction and subjective outcomes were assessed via a questionnaire at week 12.

**Results:**

21 participants completed the study. A significant reduction of acne lesions was observed at week 12 (*p* < 0.05). Treatment success rate was achieved in 61.9%, with significant improvement in PAHPI and CADI scores (*p* < 0.05). By week 12, 61.9% of participants experienced no irritation. Common side effects included dryness (81.0%), burning sensation (71.4%) and erythema (52.4%). Most participants were satisfied with the outcomes (85.7%), and willing to continue and recommend it to others (81.0%).

**Conclusion:**

Trifarotene demonstrated significant efficacy and good tolerability supporting its use as a valuable option for acne management in Malaysia.

## 1 Introduction

Acne vulgaris is one of the most common conditions seen in dermatology clinics worldwide, affecting approximately 9.4% of the global population and ranking as the eighth most common disease worldwide [1]. It usually happens in adolescents and young adults. The prevalence of acne among secondary school students in Malaysia is about 64.7% [2], aligning with two local studies among Malaysian university students with a prevalence of 60.7% to 75.8% [3, 4]. Acne vulgaris has been proven to negatively impact patients' emotions, self‐esteem, social interactions and interpersonal relationships if left untreated [5, 6]. Approximately 85% of adolescents experience both physical and emotional impacts from acne vulgaris [7].

Topical retinoids remain the mainstay of acne treatment, with options such as adapalene, tretinoin, and tazarotene available in different formulations [8]. Over the years, four generations of retinoids have been developed, each with different molecular properties and specificity for retinoic acid receptor (RAR) subtypes [9].

Trifarotene Cream 0.005% (AKLIEF), approved in October 2019, is the latest generation of topical retinoids which received Food and Drug Administration (FDA) approval [10]. Its recent approval in Malaysia in July 2024 further underscores its growing global clinical relevance. It is the only topical retinoid that binds selectively to RAR‐γ receptors, the predominant subtype in human skin [11, 12]. Notably, trifarotene modulates unique pathways beyond traditional retinoid mechanisms. By binding to RAR‐γ receptors, it initiates receptor dimerization and binds to specific retinoic acid response elements (RAREs) that regulate retinoid‐responsive genes [13]. These transcriptional modifications constitute the core mechanism underlying its comedolytic, anti‐inflammatory, and depigmenting effects. Comprehensive gene expression profiling has revealed that trifarotene modulates three key biological pathways [1] Skin hydration, in which it reinforces epidermal barrier function and hydration by upregulating peptidyl‐arginine deiminase 1 and aquaporin‐3 [2] Cell adhesion, in which by attenuating hemidesmosomal integrity, it reduces keratinocyte cohesion, facilitating comedolysis [3] Proteolysis, in which it limits collagen and elastin degradation by downregulating matrix metalloproteinases (MMPs), contributing to improved skin texture and resilience [14, 15]. These molecular effects highlight its distinct advantage over earlier‐generation retinoids.

Trifarotene has demonstrated strong anti‐comedogenic, anti‐inflammatory and anti‐pigmentation properties in vivo [15], making it a significant advancement in acne management. Pivotal and long‐term trials have shown that trifarotene is effective on facial acne with a favorable safety profile [8, 16]. In the PERFECT 1 and 2 studies, it significantly reduced facial acne lesions as early as week 1 [16]. A subsequent 52‐week trial confirmed sustained improvement in inflammatory and non‐inflammatory lesions, and patient‐reported quality of life, with IGA success rate continuing to increase beyond 12 weeks [8]. It was also proven to be well tolerated, with mostly mild‐to‐moderate local irritations peaking early at week 1 and resolving thereafter [16]. Trifarotene is the only topical retinoid that has been studied and shown to have significant efficacy for not only facial acne, but also truncal acne [17]. Beyond acne, it was found that trifarotene usage helps in early acne scar and post‐acne hyperpigmentation reduction as early as after 2 weeks of usage, with similar excellent tolerability and minimal treatment‐emergent adverse events [18]. Despite robust global data, clinical evidence in Asian populations remains limited. Hence, this study was conducted to observe the efficacy and tolerability of trifarotene in acne management over 12 weeks within the Malaysian population.

## 2 Materials and Methods

This prospective observational study was conducted at a university hospital in Malaysia, using a convenience sampling method. The study was conducted in accordance with the Declaration of Helsinki and the Malaysian Good Clinical Practice Guideline, with ethical approval from the Institutional Review Board (Reference number: JKEUPM‐2025‐174).

**FIGURE 1:**
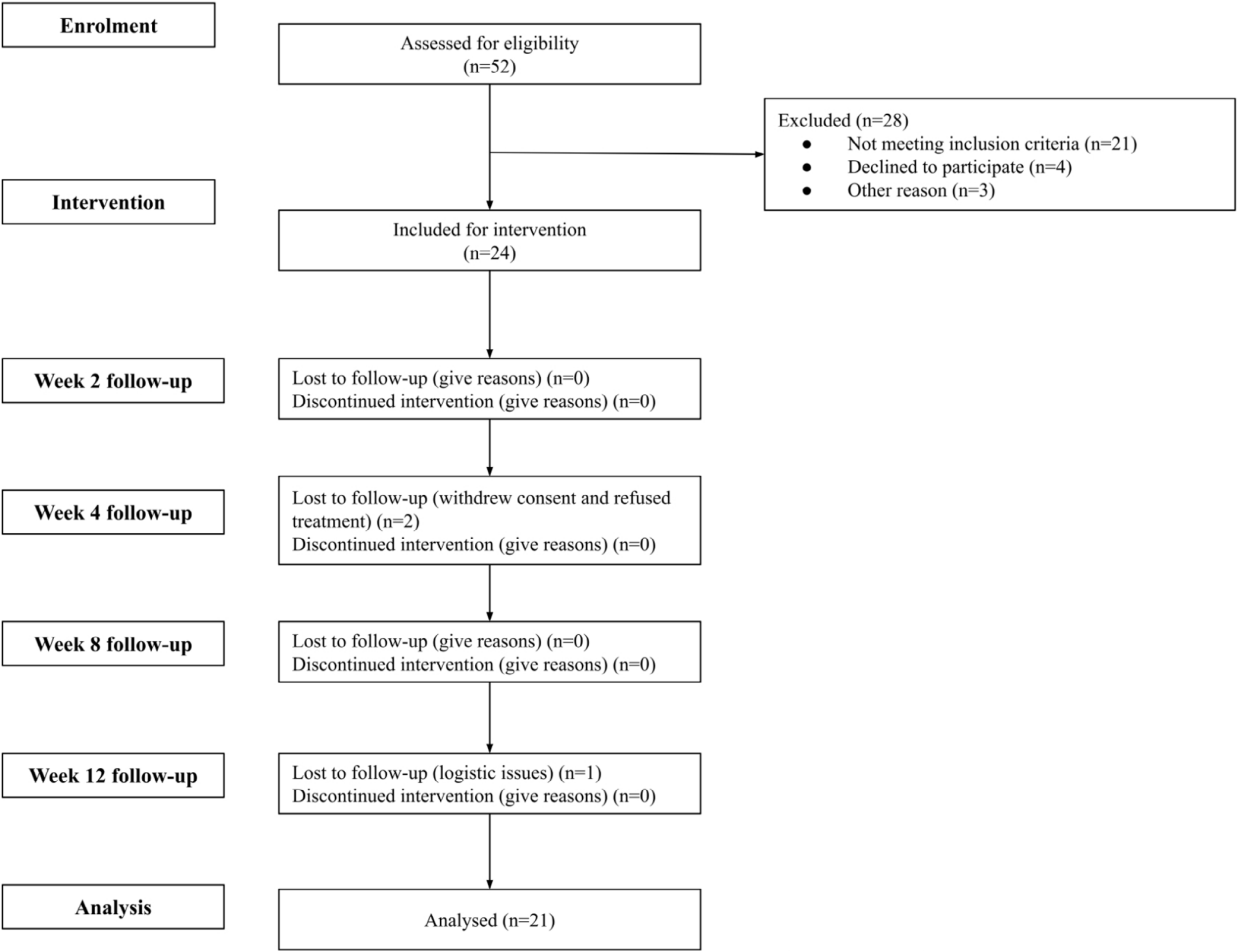
Study flow diagram. Following recruitment, 24 participants with mild to moderate facial acne were included to receive trifarotene. During the study period, three participants dropped out of the study.

A total of 52 participants who expressed interest in participating in the study were assessed for eligibility through clinical evaluation based on the predefined inclusion and exclusion criteria. Eligible participants were those with mild‐to‐moderate facial acne (Investigator's Global Assessment (IGA) score of 2 to 3) and willing to avoid excessive ultraviolet exposure. Exclusion criteria included recent use of anti‐acne therapy, severe or secondary acne, interfering dermatologic conditions or procedures, and hypersensitivity to trifarotene. Of these, 28 participants were excluded after screening. The most common reason for exclusion was attributable to a discrepancy between participants' self‐perceived acne severity and the clinically assessed IGA score, with most presented with acne severity below the required IGA score. After detailed explanation of the study, 24 participants provided written informed consent to participate (Figure 1). All assessments were performed by the principal investigator, a board‐certified dermatologist. Participants were followed for 12 weeks, with five scheduled assessments at week 0, 2, 4, 8, and 12. During the study period, three participants dropped out due to treatment refusal and logistic issues, including difficulties attending scheduled follow‐up visits. No participants discontinued due to severe adverse effects.

As part of the standardized skincare protocol, all participants were given cleanser (Cetaphil Gentle Skin Cleanser, Galderma), moisturizer (Cetaphil Moisturizing Lotion, Galderma), and sunscreen (Cetaphil Sun SPF 50+ Light Gel, Galderma). A thin layer of trifarotene was applied after cleansing the face and application of moisturizer. Participants were instructed to begin with half a pump and gradually self‐titrate up to one full pump nightly for 12 weeks. If irritation developed, they were allowed to temporarily cease treatment for two days and recommence once irritation subsided. Sun exposure should be minimized throughout the study.

Clinical and photographic assessments were conducted at every follow‐up visit. Standardized images were taken using the Lifeviz Mini Camera. Clinical evaluation was assessed using multiple validated tools, including absolute lesion counts. Facial acne severity was graded using the Investigator's Global Assessment (IGA), a 5‐point scale ranging from 0 (clear) to 4 (severe) [17]; treatment success was defined as a score of 0 or 1 and ≥ 2‐grade improvement [16]. Post‐inflammatory hyperpigmentation was measured via the Post‐Acne Hyperpigmentation Index (PAHPI), a validated clinical scoring system designed to quantify the extent and intensity of hyperpigmented lesions following acne. The PAHPI evaluates three key parameters [1] the number of hyperpigmented lesions [2] the intensity of pigmentation, and [3] the anatomical distribution of lesions across the face. Each parameter is scored separately, and the total PAHPI score is calculated as the sum of individual component scores, with higher scores indicating greater severity of post‐acne hyperpigmentation. The total score ranges from a minimum of *6* to a maximum of *22* points [19]. Quality of life impact was assessed using the Cardiff Acne Disability Index (CADI), a 5‐item scale yielding a total score of 0–15, where higher scores indicate greater impairment [20]. Additionally, local cutaneous tolerability was assessed at each visit using a modified 4‐point safety assessment scale adapted from Leyden et al. (2009) [21]. The scale evaluated erythema, dryness, scaling and burning or stinging sensation, with scores ranging from 0 “none” to 3 “severe”, where higher scores indicate greater severity of local skin reactions. A participant satisfaction questionnaire was completed at week 12 to document subjective outcomes.

Data analysis was performed using IBM Statistical Package for Social Science (SPSS) version 29.0. For descriptive analysis, all continuous independent variables were tested for normality testing and the results were reported in mean ± standard deviation (SD). Categorical variables were summarized as frequency and percentage. Linear Mixed Model (LMM) was used to evaluate the changes in outcome measures over time and within‐subject variability. Post hoc pairwise comparisons were conducted with Bonferroni correction to adjust for multiple testing. *p*‐value < 0.05 was considered statistically significant.

## 3 Results

### 3.1 Participants Characteristics

A total of 21 participants were included for final analysis, with a mean age of 22.4 ± 1.6 years. The cohort was predominantly female (61.9%) and Malay (66.7%) participants. Sociodemographic and baseline disease characteristics are summarized in Table 1.

**TABLE 1:** Sociodemographic and baseline disease characteristics.

Variables	Mean ± SD/*n* (%)
**Sociodemographic**	
Age, years	22.4 ± 1.63
Gender	8 (38.1)
Male	13 (61.9)
Female	
Ethnicity	14 (66.7)
Malay	6 (28.6)
Chinese	1 (4.7)
Indian	
**Baseline Disease**	
Inflammatory Lesion Counts	17.9 ± 12.93
Non‐inflammatory Lesion Counts	32.5 ± 24.55
IGA Score	15 (71.4)
2 (Mild)	6 (28.6)
3 (Moderate)	
PAHPI Score	10.0 ± 3.63
CADI Score	6.9 ± 2.33

### 3.2 Efficacy

**FIGURE 2:**
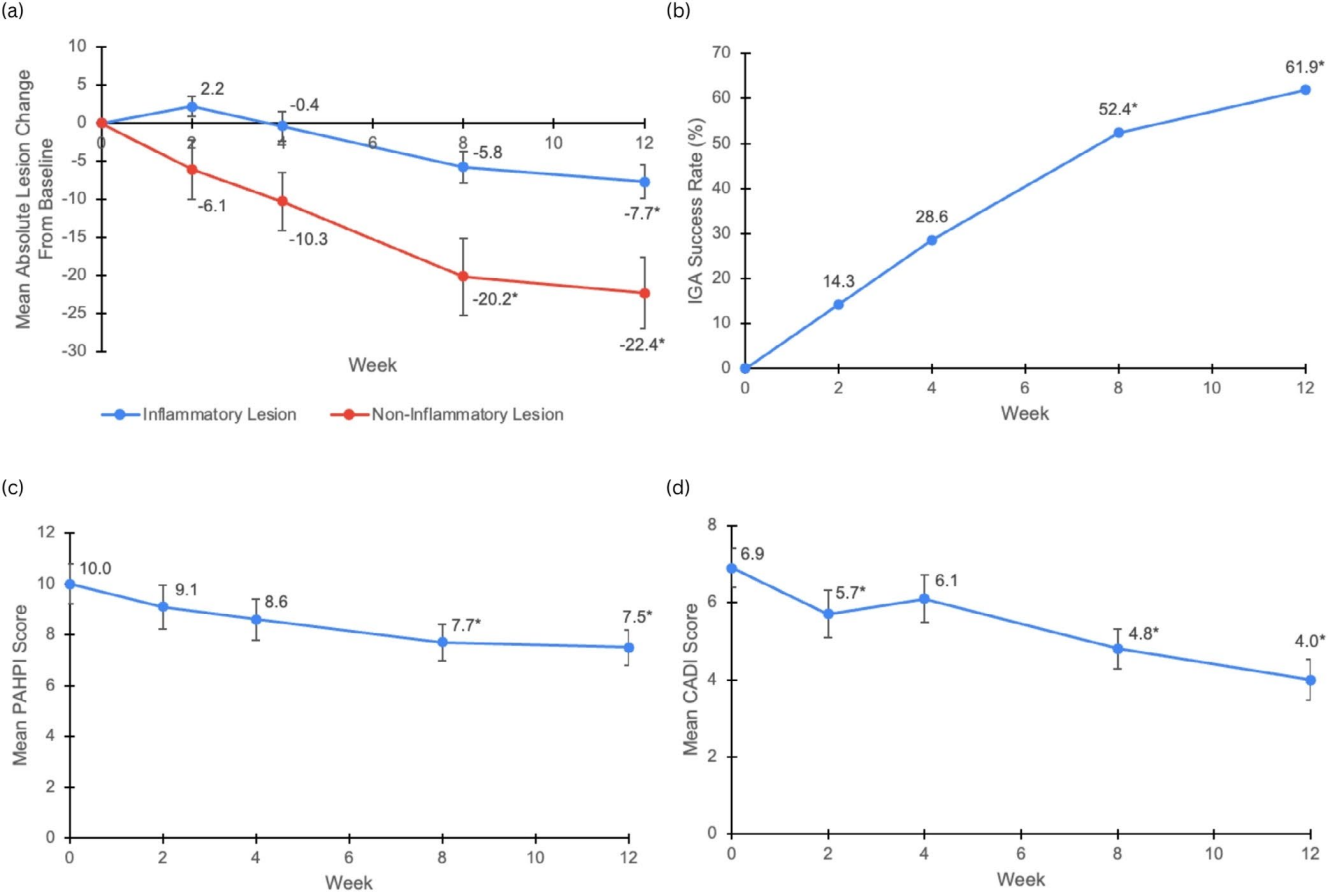
Efficacy of Trifarotene Over 12 Weeks. (a) Mean absolute change in lesion from Baseline, showing the reduction in inflammatory and non‐inflammatory lesion counts. (b) Investigator's Global Assessment (IGA) success rate. (c) Mean Post‐Acne Hyperpigmentation Index (PAHPI) score. (d) Mean Cardiff Acne Disability Index (CADI) score; Error bars represent standard error; * = statistically significant changes from baseline (*p* < 0.05).

**FIGURE 3:**
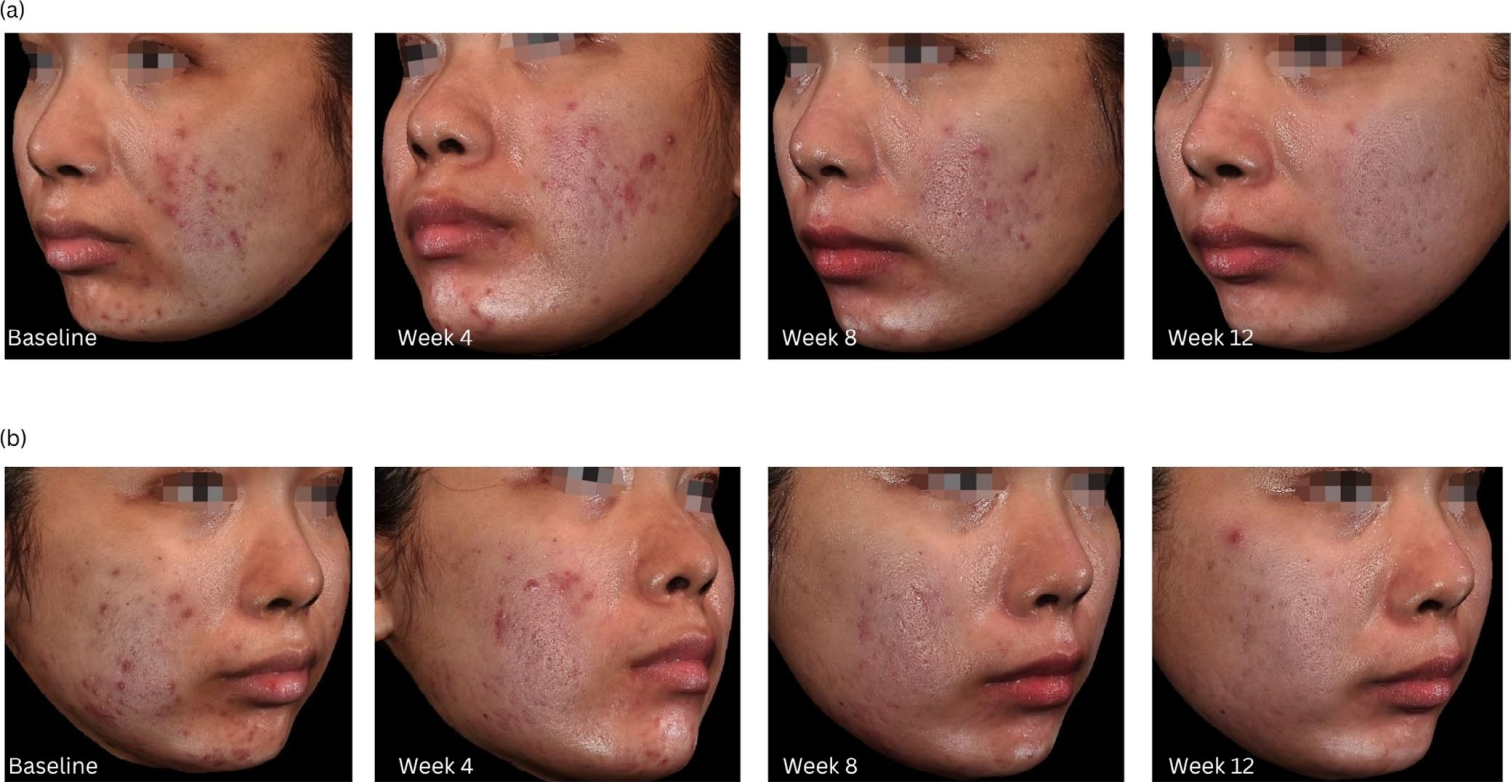
Representative Clinical Photographs of Participant 1 Over 12 Weeks. (a) Left side. (b) Right side.

**FIGURE 4:**
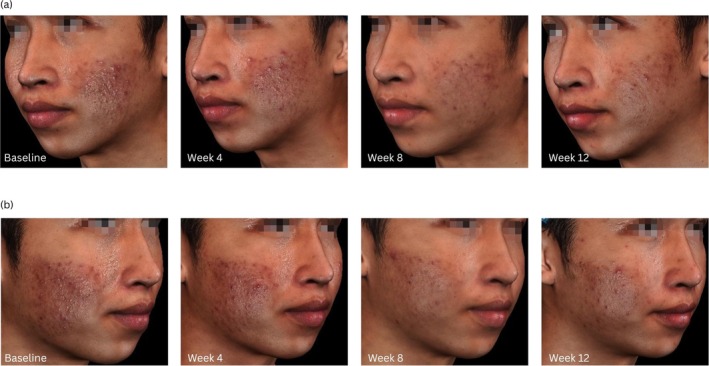
Representative Clinical Photographs of Participant 2 Over 12 Weeks. (a) Left side. (b) Right side.

**FIGURE 5:**
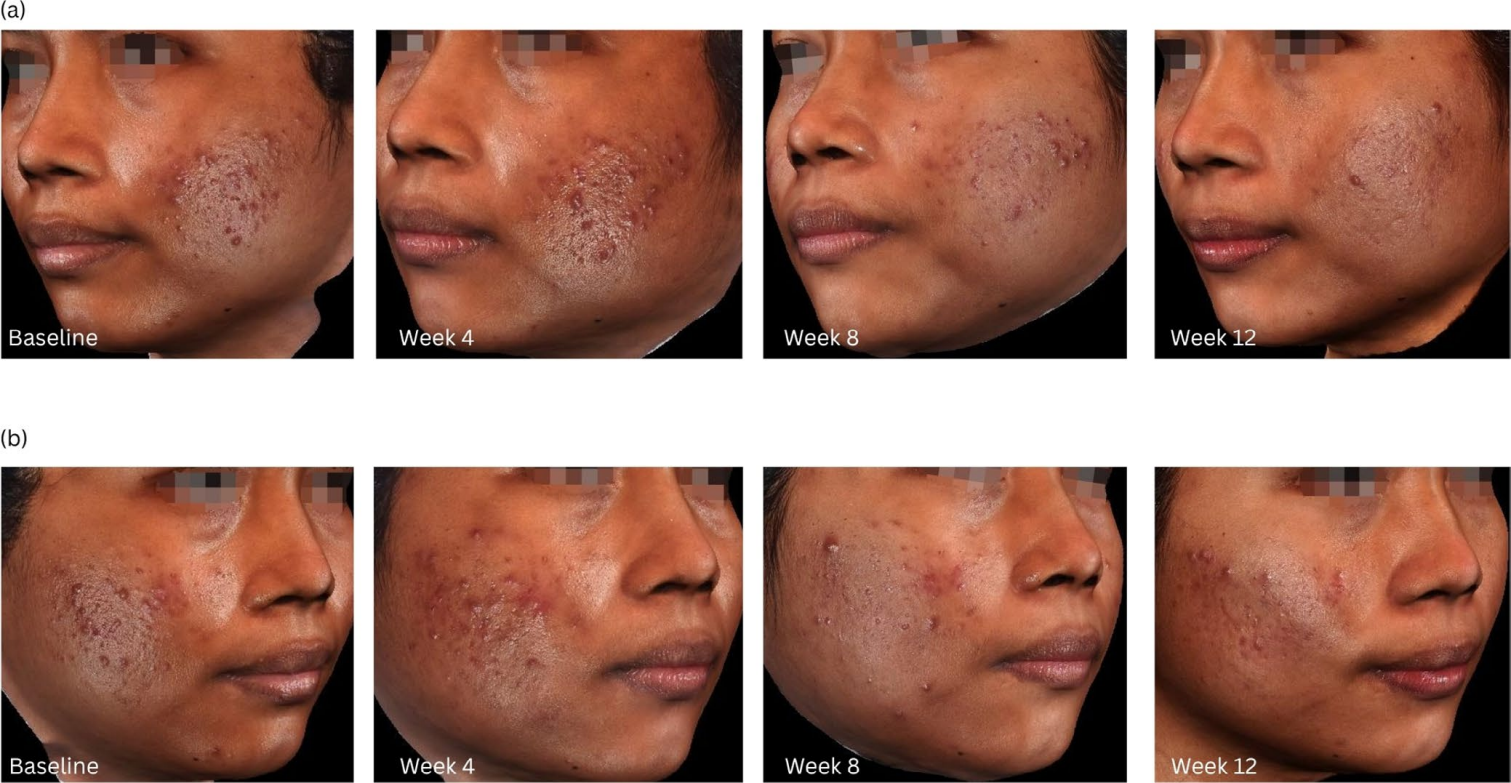
Representative Clinical Photographs of Participant 3 Over 12 Weeks. (a) Left side. (b) Right side.

Figure 2 illustrates the clinical efficacy of trifarotene over 12 weeks, including changes in mean inflammatory and non‐inflammatory lesion counts, IGA success rates, PAHPI and CADI scores. The representative clinical photographs demonstrating treatment progress are provided in Figures 3, 4, 5.

#### 3.2.1 Absolute Inflammatory Lesion Counts

Trifarotene produced a statistically significant reduction in inflammatory lesion counts at week 12 compared with baseline (*p* < 0.05). A transient mean increase of 2.2 lesions was noted at week 2, followed by a progressive decline thereafter, reaching a mean reduction of 7.7 lesions by week 12.

#### 3.2.2 Absolute Non‐Inflammatory Lesion Counts

A statistically significant decrease in non‐inflammatory lesion counts was observed from week 8 onwards, in comparison to baseline (*p* < 0.05). By week 12, the mean lesion counts had decreased by 22.4.

#### 3.2.3 Investigator's Global Assessment (IGA)

The proportion of participants achieving treatment success increased steadily throughout the study, reaching 61.9% at week 12. Statistically significant improvement was observed at both week 8 and week 12, as compared to baseline (*p* < 0.05).

#### 3.2.4 Post‐Acne Hyperpigmentation Index (PAHPI)

Mean PAHPI score declined from 10.0 at baseline to 7.5 at week 12, demonstrating a significant improvement at week 8 and week 12 in comparison to baseline (*p* < 0.05).

#### 3.2.5 Cardiff Acne Disability Index (CADI)

Mean CADI score decreased from 6.9 at baseline to 4.0 at week 12, with a transient rise at week 4 (mean 6.1). Significant improvements were recorded from week 2 onward (*p* < 0.05), as compared to baseline except for week 4 (*p* = 0.60).

### 3.3 Tolerability

**FIGURE 6:**
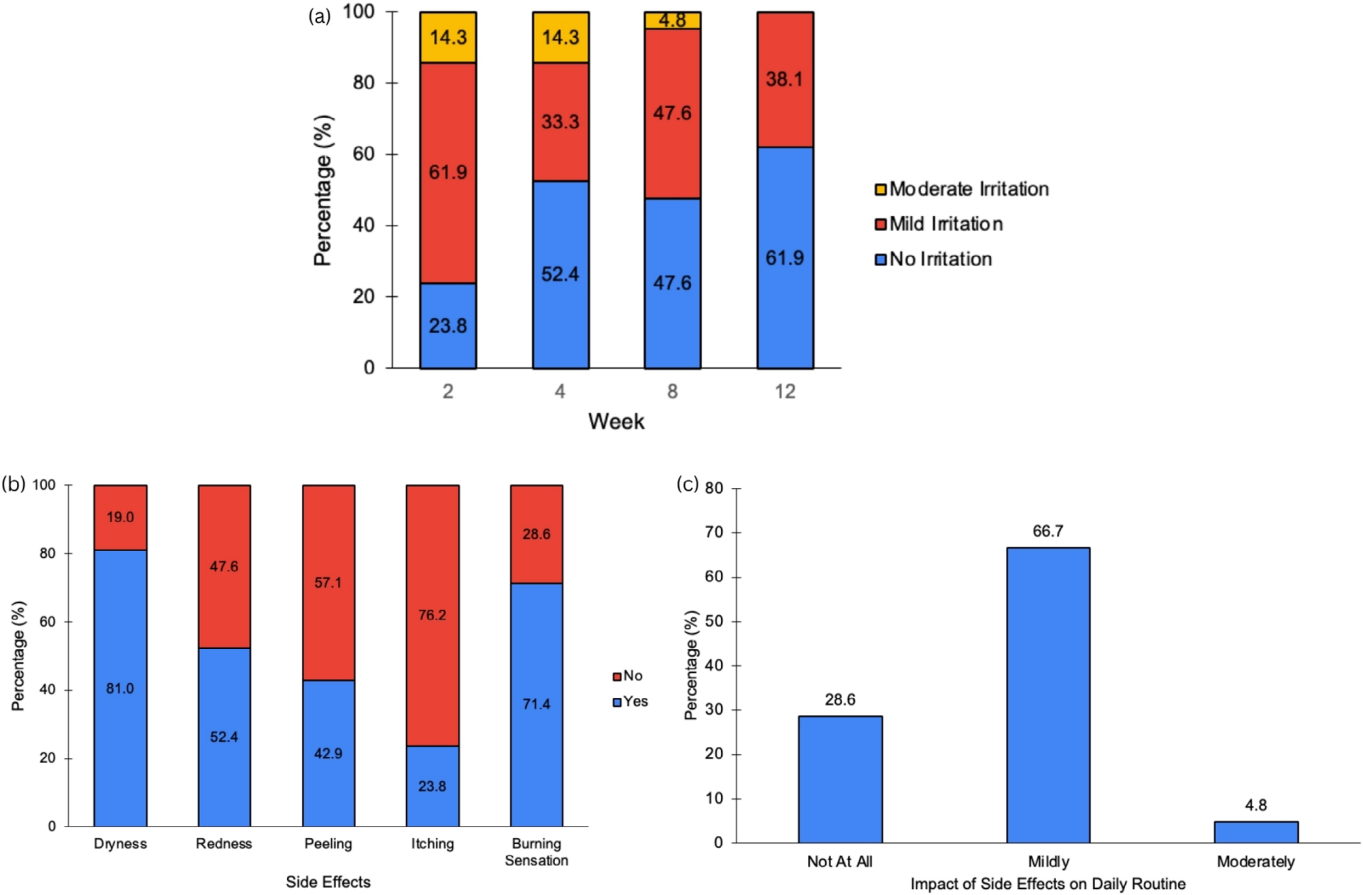
Tolerability of Trifarotene Over 12 Weeks. (a) Severity of local cutaneous irritation based on modified safety assessment scale. (b) Occurrence of specific side effects throughout 12 weeks. (c) Impact of side effects in daily routine.

Figure 6 summarizes the tolerability outcomes over 12 weeks. At week 2, most participants (76.2%) experienced mild‐to‐moderate irritation based on the modified safety assessment scale. By week 12, tolerability had improved substantially, with 61.9% experiencing no irritation.

The most commonly self‐reported side effects were dryness (81.0%), burning sensation (71.4%) and redness (52.4%). However, the vast majority of participants reported either no (28.6%) or mild (66.7%) impact of side effects on their daily lives, with just 4.8% experiencing moderate disruption.

### 3.4 Participant Satisfaction

**FIGURE 7:**
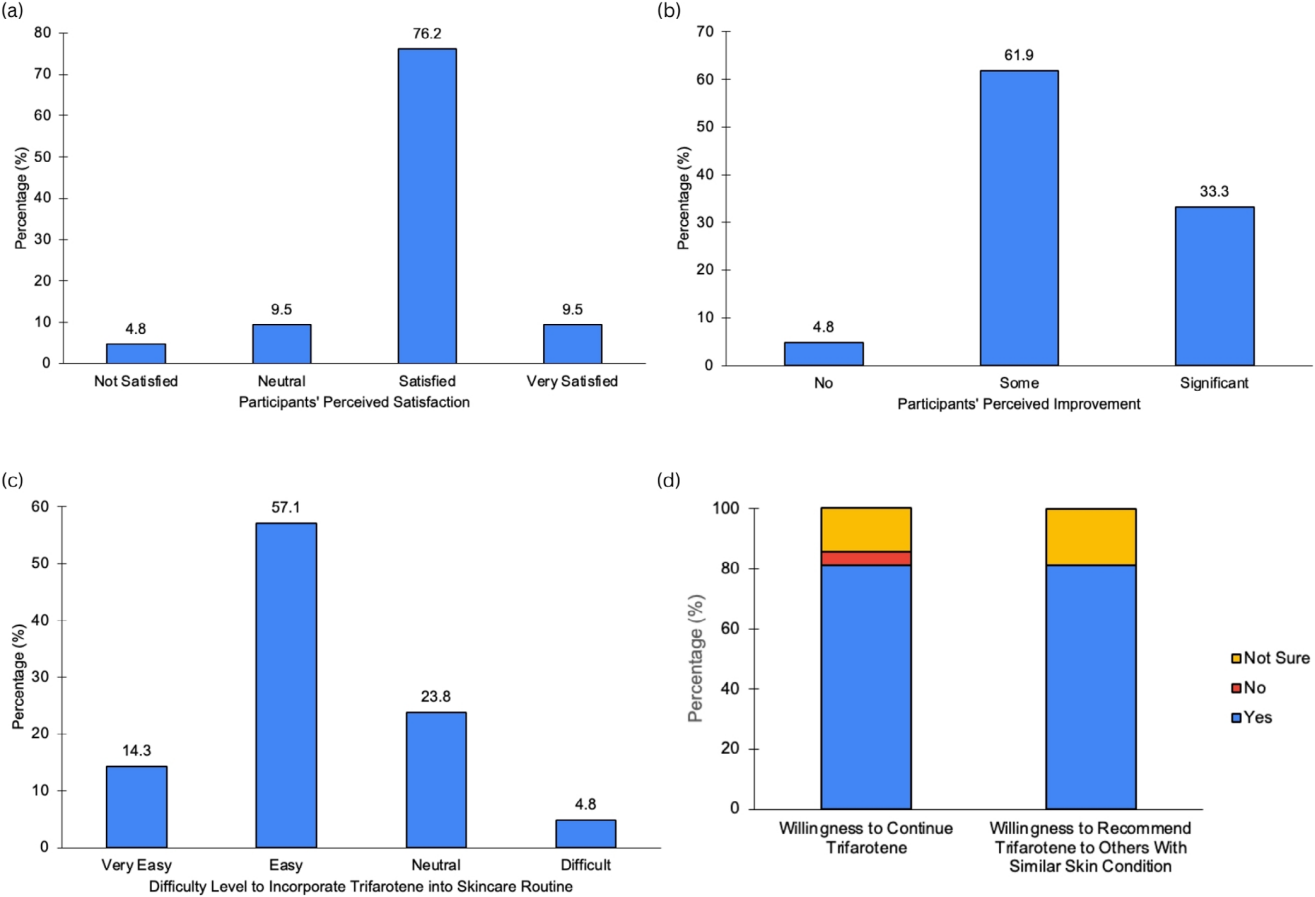
Participants' Satisfaction and Treatment Perception. (a) Perceived satisfaction with trifarotene. (b) Perceived overall improvement. (c) Difficulty in incorporating trifarotene into skincare routine. (d) Willingness to continue treatment and recommend trifarotene to others with similar skin conditions.

As shown in Figure 7, participant satisfaction was high. By week 12, 85.7% of participants reported being satisfied or very satisfied with treatment outcomes and 95.2% perceived some or significant visible improvement. Trifarotene was rated as easy or very easy to incorporate into daily skincare routines (71.4%). The majority (81.0%) indicated willingness to continue therapy and to recommend it to others with similar skin conditions.

## 4 Discussion

Our study demonstrated that trifarotene produced significant clinical improvements in acne severity over 12 weeks, with notable reductions in both inflammatory and non‐inflammatory lesion counts, aligned with several global studies [8, 16, 19]. A transient increase in inflammatory lesions observed at week 2 reflected the well‐recognized “retinoid flare” phenomenon, characterized by early exacerbation of inflammation before clinical improvement. This could be due to retinoid‐induced exacerbation of inflammation, the natural progression of acne overriding therapeutic effects, or a delayed onset of treatment response [22]. This finding, though not previously highlighted in global studies, was consistently observed in our study population. Participants should be pre‐warned about this possible side effect to ensure that the full therapeutic potential of the drug can be properly evaluated. Early termination of treatment may otherwise prevent the true efficacy from being reflected. While reassurance may be sufficient in most cases, the side effect can also be managed concurrently with benzoyl peroxide or doxycycline, particularly in patients who are more anxious. A global study further demonstrated that the combination of trifarotene and oral doxycycline has significant efficacy and a favorable safety profile in patients with severe acne [23].

Inflammatory lesions in our cohort improved more slowly than comedonal lesions, mirroring findings from the PERFECT trials, in which non‐inflammatory lesions demonstrated earlier and greater reductions [16]. Retinoids primarily normalize follicular keratinization and comedogenesis, which is why they remain the first‐line therapy in current Asia‐Pacific recommendations [24] Hence, resolution of inflammatory lesions requires additional down‐regulation of cytokines, such as IL‐1β, TNF‐ɑ, and IL‐17 [25]. Consequently, longer treatment durations may be needed for optimal clearance. Transcriptomic analysis by Dréno et al. (2021) revealed that trifarotene modulates a unique set of genes specifically involved in cellular migration and inflammation. By downregulating pathways required for leukocyte recruitment and promoting extracellular matrix reorganization, trifarotene effectively reverses the underlying molecular drivers of acne inflammation [26].

Investigator's Global Assessment (IGA) success rates improved progressively and reached statistical significance from week 8 onwards, aligning with the PERFECT 2 trial [16]. However, the success rate at week 12 in our study (61.9%) was higher than those reported internationally (29.4%–42.3% in PERFECT trials; 26.6% in other global cohorts) [8, 16]. This finding further supports the results of global studies, reaffirming the efficacy of trifarotene in the management of acne vulgaris. Although statistically significant improvements were not evident during the early phase, the final endpoint outcomes surpassed those of previous reports. A possible explanation lies in the study methodology, which encouraged subjects to gradually up‐titrate the application frequency until reaching their maximum level of tolerability.

Interestingly, our study showed a significant improvement in post‐acne hyperpigmentation, with PAHPI scores declined at week 12. The Asia‐Pacific Phase IV LEAP study further substantiated these findings, showing that trifarotene significantly reduced post‐inflammatory hyperpigmentation (PIH) and overall disease severity (ODS) by week 12 compared with vehicle. Moreover, continued improvement in Post‐Acne Hyperpigmentation Index (PAHPI) scores was observed through week 20 and 24, highlighting its sustained efficacy over time [24]. Mechanistically, retinoids are known to increase epidermal turnover, inhibit tyrosinase and related enzymes, reduce melanosome transfer, and facilitate melanin dispersal, which can contribute to early fading of macular hyperpigmentation [24]. Moreover, the concurrent use of sunscreen and moisturizer in our study may have contributed to the rapid reduction in hyperpigmentation. This observation is supported from a recent systematic review, which highlighted that treatment‐induced pigment clearance is highly influenced by adjunctive photoprotection. Consistent use of sunscreen and moisturizer was shown to accelerate the fading of hyperpigmentation [27].

Our study cohort demonstrated a consistent improvement in CADI score from baseline to week 12, indicating a meaningful reduction in acne‐related psychosocial burden. Improvements were apparent as early as week 2 and most pronounced at week 12, which aligns with findings from a real‐world study [8]. Interestingly, a transient increase in CADI scores at week 4, which may be attributed to retinoid dermatitis [24], activation of underlying inflammatory pathways in acne [25], or the transient flare of inflammatory lesions commonly seen during the initial phase of retinoid therapy due to “retinoid flare” [5]. This temporary clinical worsening can negatively affect patients' self‐perception and quality of life, potentially leading to early discontinuation of therapy. Such premature cessation may prevent the full therapeutic potential of trifarotene from being realized, underscoring the importance of patient education and reassurance during this phase.

Our cohort demonstrated a higher incidence of cutaneous adverse events, including erythema, dryness, and burning sensations compared with the PERFECT trials [16]. Several factors may explain this observation. First, our study population consisted exclusively of Malaysian participants, representing an Asian demographic with inherently higher baseline skin sensitivity. Previous studies have shown that Asian skin types are more susceptible to retinoid‐induced irritation due to differences in epidermal barrier integrity and stratum corneum lipid composition, which predispose to increased transepidermal water loss and erythema [26, 27]. This contrasts with the predominantly Caucasian populations included in earlier global trials. In addition, Malaysia's tropical climate, characterized by persistent heat and humidity, may have further intensified local irritation and amplified the perception of discomfort.

The local irritation observed in our participants likely reflects retinoid dermatitis, a common and transient effect related to accelerated epidermal turnover and temporary barrier disruption [28]. Although generally self‐limiting, such reactions remain a key reason for treatment discontinuation, particularly among Asian patients [29]. To improve tolerability, therapy should be initiated gradually, using a lower concentration or applied on alternate days for the first two weeks. Supportive skincare practices, such as gentle cleansing and the use of moisturizers applied either 10 min before or mixed with the retinoid, can further reduce irritation and prevent post‐inflammatory hyperpigmentation [30, 31, 32]. Moreover, comprehensive patient education on correct application techniques, expected side effects, and the importance of adherence has been shown to markedly enhance treatment persistence and satisfaction [33, 34].

Notably, despite the higher incidence of cutaneous side effects, patient satisfaction in our study remained high. 85.7% of participants reported being satisfied with their treatment outcomes, 95.2% perceived visible improvement, and 71.4% found trifarotene easy to incorporate into their daily skincare routine. Furthermore, 81.0% expressed willingness to continue therapy and to recommend it to others with similar skin conditions. These findings highlight that, with appropriate counseling and supportive measures, trifarotene remains well‐tolerated and positively received even among populations with higher baseline skin sensitivity.

Our study has several notable strengths. First, it prospectively evaluated the efficacy and tolerability of topical trifarotene in a real‐world population of patients with mild‐to‐moderate acne, with systematic follow‐ups. Our findings will provide valuable preliminary insights and serve as foundational data for future research in Malaysia. Second, we incorporated not only clinical outcomes such as absolute lesion counts and IGA, but also participant‐reported outcomes, including satisfaction on the effectiveness of trifarotene, which allowed a more holistic evaluation of treatment response.

Nonetheless, certain limitations should be acknowledged. The study was conducted with a relatively small sample size, which may have limited the statistical power to detect subtle differences and generalisability of data. The study duration was restricted to 12 weeks, which may not fully capture the long‐term benefits of trifarotene or relapse rates following treatment cessation.

In conclusion, trifarotene demonstrated favorable efficacy and acceptable tolerability within our study cohort. Although local cutaneous adverse events were observed, these did not compromise patient satisfaction, willingness to continue therapy, or the ease of integrating the treatment into daily skincare routines.

## Author Contributions

**Kang Nien How:** conceptualization (lead); investigation (lead); methodology (lead); supervision (lead); writing – review and editing (equal). **Jun Hui Goh:** formal analysis (supporting); methodology (equal); writing – original draft (lead). **Tatt Quan Tan:** formal analysis (lead); methodology (equal); writing – original draft (supporting). **Wen Chung Ho:** conceptualization (supporting); investigation (supporting); writing – review and editing (equal). **Chi Fai Lee:** investigation (supporting); writing – review and editing (equal). **Meroshini Sundaran:** methodology (supporting); writing – review and editing (equal). **JiaXu An:** formal analysis (supporting); writing – original draft (supporting).

## Ethics Statement

This study was approved by the Institutional Review Board (IRB) of Universiti Putra Malaysia (UPM), Ethical Committee for Research Involving Human Subjects (Reference Number: JKEUPM‐2025‐174). This prospective observational study was conducted at Hospital Sultan Abdul Aziz Shah (HSAAS) Malaysia using a convenience sampling method.

## Consent

Written informed consent was obtained from all participants for publication of their clinical information and images. The participants were informed that their identities would keep anonymous to preserve their confidentiality.

## Conflicts of Interest

The authors declare no conflicts of interest.

## Data Availability

The data that support the findings of this study are available from the corresponding author upon reasonable request.
